# Particulate accumulated matter as an indicator of coastal benthic habitat condition

**DOI:** 10.1007/s13280-025-02249-y

**Published:** 2025-10-11

**Authors:** Louise Forsblom, Antti Takolander, Anu Kaskela, Markku Viitasalo, Elina A. Virtanen

**Affiliations:** 1 Finnish Environment Institute (Syke), Helsinki, Finland; 2 Geological Survey of Finland (GTK), Espoo, Finland; 3 University of Helsinki, Helsinki, Finland

**Keywords:** Condition indicator, Eutrophication, Marine habitats, Particulate accumulated matter, Restoration, Sedimentation

## Abstract

Eutrophication adversely affects coastal areas, but most indicators of eutrophication demonstrate temporal or large-scale changes in eutrophication. These usually cannot be used in local actions, such as restoration or conservation. Eutrophication also results in increased accumulation of particulate matter and here we assess the usefulness of particulate accumulated matter (PAM) as an indicator for marine habitat condition in the northern Baltic Sea. We use > 50 000 observations of PAM, to spatially model the accumulation of PAM at shallow benthic substrates, and subsequently demonstrate the use of PAM as an indicator for benthic habitat quality*.* Predictions show only 4.8% of the shallow marine area as not affected by PAM and the abundance of PAM negatively affected the occurrence of *Fucus* habitats. Estimates of amount of PAM can be used for determining areas that will benefit from restoration and nutrient abatement actions and for locating the most pristine areas in need of conservation action.

**Supplementary Information:**

The online version contains supplementary material available at 10.1007/s13280-025-02249-y.

## Introduction

Anthropogenically induced eutrophication is of large concern in coastal areas and causes algal blooms, oxygen depletion, and habitat degradation, severely impacting coastal ecosystems (Burkholder et al. 2007; Carstensen et al. 2014; Hou et al. 2022). Eutrophication is usually defined as excessive anthropogenic input of nutrients and their effects in aquatic ecosystems (Andersen et al. 2006). It is one of the main environmental problems of the Baltic Sea (Andersen et al. 2017), driven by excessive nutrient loads and amplified by the recent warming of surface waters (Safonova et al. 2024). The settling organic matter serves as an essential food source for deposit-feeding benthic infauna and surface-feeding invertebrates (Griffiths et al. 2017), but particles in the water or on the surface of vegetation reduce the amount of light available for photosynthesis, impacting benthic photoautotrophs (Krause‐Jensen and Sand‐Jensen 1998). Increased sedimentation and organic matter deposition can also negatively impact benthic habitats (Harrington et al. 2005; Stender et al. 2014), and, e.g., when the substrate is covered by particulate matter, the attachment of algal zygotes and mussel larvae is hindered, which affects colonization of the species (Eriksson and Johansson 2003).

Semi-enclosed sea areas, such as the Baltic Sea, are particularly sensitive to increased sedimentation, caused by eutrophication and potentially also climate change (Viitasalo and Bonsdorff 2022). In extreme cases, increased sedimentation of organic matter coupled with reduced mixing can lead to hypoxia, both in deep basins and in shallower archipelagos (Carstensen et al. 2014; Virtanen et al. 2019), leading to so-called dead zones (Breitburg et al. 2018). Organic matter that accumulates in deeper sediments primarily originates from sedimentation of phytoplankton, with sedimentation rates being modified by consumers and decomposition (Turner 2002; De La Rocha and Passow 2007; Spilling et al. 2018). In coastal areas, this organic matter can also have an allochthonous origin, being flushed from land to sea through surface runoff and river discharge (Bianchi 2011). River water can also carry significant amounts of inorganic particles, particularly during spring floods, when large quantities of clay and sand are transported from the watershed into the sea (Syvitski et al. 2005). In areas where currents and water mixing reach the seafloor, sediments may be resuspended into the water column. In such regions, the bottom sediments can be transported both laterally and vertically, depending on the strength and direction of currents and the local topography (Ran et al. 2022).

Current studies within the Baltic Sea environment focus on processes involved in the sinking, transport, resuspension, and deposition of the sedimentary material and organic matter (e.g., Christiansen et al. 2002; Emeis et al. 2002; Kuhrts et al. 2006; Danielsson et al. 2007; Seifert et al. 2009; Almroth-Rosell et al. 2011; Griffiths et al. 2017). Several of these studies also discuss the “fluffy layer,” consisting of unconsolidated loose material accumulating at the sediment–water interface. Besides sedimentation processes, there are studies on the characteristics of sedimentary organic matter (Winogradow and Pempkowiak 2018) and on the factors influencing fluffy layer suspended matter (Pempkowiak et al. 2005), among others. Some Baltic Sea-scale spatial studies, which have focused on identifying particulate organic carbon distribution in surface sediments and sedimentation areas based on sedimentation rates from long-term deposition areas, exist (Leipe et al. 2011; Mitchell et al. 2021). However, these studies do not focus on habitat conditions, nor do they consider short-term accumulation, and few studies report spatial patterns in sedimentation.

Several indicators have been developed to monitor eutrophication status and to set reference levels to reach good ecological status, as guided by different EU policies, such as the Marine Strategy Framework Directive (MSFD) and Water Framework Directive (WFD). These include indicators such as nutrient concentrations (P and N), chlorophyll-*a*, water transparency, and the extent of hypoxic zones. While these indicators are valuable for reporting the eutrophication status at regional or basin scale and often have high temporal resolution, they cannot necessarily be used for ecological status assessments on the scale of benthic coastal habitats or habitat patches, thus limiting our understanding of localized impacts of eutrophication processes that directly impact habitats and species (Eriksson and Bergström 2005; Burkholder et al. 2007). For benthic habitat types, habitat condition is often assessed based on abiotic indicators (Kontula and Raunio 2019), as directly monitoring the effects of pressures such as eutrophication-induced burial is challenging. To target mitigation actions in support of both restoration and conservation, more detailed information to support the assessment of the secondary impacts of eutrophication on benthic habitat condition is needed.

Here we assess the usefulness of particulate matter accumulating in the fluffy surface layer on the seabed as an indicator of benthic habitat condition. Following the definition by Witt et al. (2001), the fluffy layer material is the young and mobile layer of particulate matter that accumulates on the sediment surface under quiescent conditions. The layer is water-rich and usually few centimeters thick and can be composed of organic and inorganic material. We specifically investigate the drivers influencing the accumulation of particulate accumulated matter (PAM) and its distribution as well as use the distribution of habitat-forming species *Fucus vesiculosus* as an example to demonstrate the potential of PAM as an indicator for benthic habitat quality.

## Materials and methods

### Study area and key study species

The study area is located in the northern Baltic Sea and covers the Finnish Sea area. The northern Baltic Sea is a shallow brackish water basin with steep salinity gradient and a complex archipelago with numerous islands. The area is influenced by multiple rivers, and all basins suffer from anthropogenically induced eutrophication (Andersen et al. 2017). As low salinity limits the occurrence of marine species, the vegetation on hard substrates is composed of relatively few macroalgal species. Ecologically the most important habitat-forming species is the bladderwrack (*Fucus vesiculosus,* hereafter *Fucus*), the only large, perennial algae in the northern Baltic Sea, which creates structurally complex habitats important for associated algae and invertebrates (Wikström and Kautsky 2007; Schagerström et al. 2014; Blanc et al. 2023). Previously *Fucus radicans* has also been identified from the area, but has recently been shown to be a clone of *Fucus vesiculosus* (Pereyra et al. 2025). *Fucus* has suffered severely from eutrophication during the twentieth century (Torn et al. 2006; Rinne and Salovius-Laurén 2020; Sahla et al. 2020). Increased turbidity has rendered the deeper bottoms unsuitable for *Fucus* due to light limitation (Bäck and Ruuskanen 2000), and the increased sedimentation, driven by eutrophication, has been shown to mechanically hamper the settlement of *Fucus* gametes on surfaces (Eriksson and Johansson 2003).

### Data

**Fig. 1:**
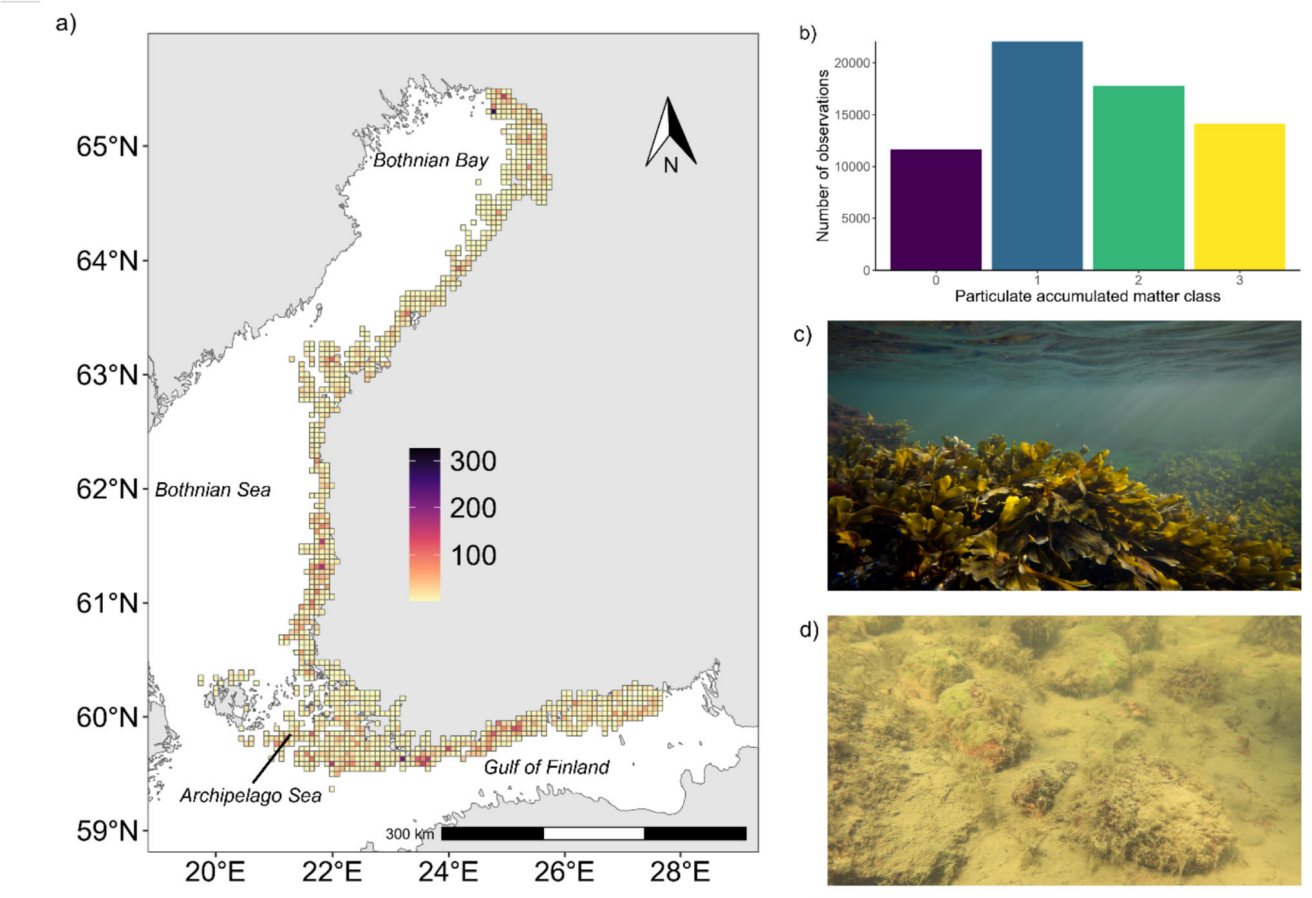
**a** Map showing the number of particulate accumulated matter observations per 5 × 5 km grids, **b** the number of observations per PAM category. Photographs show situations with no PAM **c** and with substantial amounts of PAM **d**, corresponding to classes 0 and 3, respectively. Photographs: Juuso Haapaniemi/ Parks and Wildlife Finland (**c**) and Parks and Wildlife Finland (**d**)

Biological data have been collected for the past 20 years by the Finnish Inventory Programme for Underwater Marine Diversity (Velmu, according to its Finnish acronym). The mapping efforts have mainly used either diving or video methods and focused on recording all observed macroscopic species, either as presence or as percentage cover (Forsblom et al. 2024). At the same time as the species are recorded, the abundance of particulate matter in the fluffy surface layer is also estimated, classified into four categories: 0 = no accumulation; 1 = small amount, no accumulation on vegetation but small amount on horizontal surfaces; 2 = intermediate amount, accumulation is visible on horizontal surfaces and clearly wells up; accumulation is also visible on vegetation; and 3 = substantial amounts, accumulation is very abundant and clearly covers all surfaces (Fig. 1). In the present study we use 52 535 observations of PAM, mainly collected from shallow areas with 90% of the observations collected from less than 14 m. In the shallow areas, the observations were mainly collected by diving or the use of aquascope (*N* = 38 897) and the deeper observations are mainly collected using drop video (*N* = 13 638).

### Particulate accumulated matter model

We used the Random Forest ensemble learning algorithm to model the distribution of the four classes of PAM (no accumulation, small, intermediate, and substantial amount) based on 52 535 observations (Breiman 2001), and we subsequently used the model to produce a spatial prediction of the classes across the Finnish Sea area at a 20 × 20 m resolution. The analyses were performed using the R package “randomForest” (Breiman et al. 2022; R Core Team 2022). As explanatory variables, we used morphology and complexity of the seafloor, together with variables describing the water column and sea surface characteristics. To describe openness and how prone the area is to wave disturbance, we used the share of sea (percentage of sea to land in 10 km radius), depth-attenuated exposure, as well as the average and maximum seafloor fetch. We also included bathymetry and slope in the model. We hypothesized that sheltered depressions and flat areas would have higher prevalence of PAM, while exposed and rugged seafloors would have less. Using Benthic Terrain Modeler (v3.0) (Walbridge et al. 2018), we developed two geomorphological metrics that describe the complexity of the seafloor: bathymetric position index (BPI) and vector ruggedness. BPI is a measure of surfaces higher (positive values) or lower (negative values) than their surrounding areas, representing crests and topographical depressions, while values close to zero show flat areas. We used four separate position indices with various search radii, ranging from 120 to 2 km. Vector ruggedness describes the rugosity of the seabed as the three-dimensional orientation of the grid cells within a neighborhood. We used turbidity to identify areas of lower water clarity, and distance from river outlets multiplied with river discharge, to characterize river plumes, with potentially high PAM. We also included substrate class in the model, with five levels corresponding to the folk5 classification used by EMODnet (Kaskela et al., 2019): rock and boulders, coarse substrate, mixed substrate, mud to muddy sand and sand. The substrate classes are based on prediction from presence absence models for each of the substrate classes fit using Boosted Regression Trees (Elith et al. 2008) and combined to one variable. More in-depth descriptions of the variables can be found in Takolander et al. (2025) and in Virtanen et al. (2018, 2019, 2024).

Random Forest models are built using multiple decision trees that are subsequently combined to a final model (Breiman 2001). A smaller set of random variables are selected for inclusion in each tree based on the number defined in the model function mtry. We used 20% of the data to select the optimal number of variables for mtry using the function RFtune that selects the optimal number based on bootstrap, setting mtry to 5. We validated the model using tenfold cross-validation using the remaining 80% of the data, calculating overall accuracy for the model, kappa, and class-specific precision, recall, F1 scores as well as receiver operator curves (ROC), plotting the true positive rate against the false positive rate. Accuracy measures how correct the model is overall, kappa how well the model matches the validation data, precision measures how accurate the model is in predicting occurrences of each PAM class correctly and recall how many of the true positives the model identifies, whereas the F1 score is a measure of model performance that combines both precision and recall. We used the mean decrease in Gini importance that measures the decrease in impurity attributed to a specific feature, for comparing the relative importance of the explanatory variables, measuring how much the model improves across all trees.

We also compared the predictions to an independent dataset describing sedimentation rates (EMODnet Geology 2019). The information on sedimentation rates for recent sediments is presented as point-source information focusing on the present-day sedimentation (since AD 1900 or so). The sedimentation rates estimations (centimeters/year) can be based on established historical records of anthropogenic radionuclides (e.g., 137Cs and 241Am). We used data from 149 location at depth of maximum 50 m and compared the results to the predictions from the model of PAM on surfaces. We further investigated how the spatial predictions of PAM classes correspond to the ecological status assessed in the Water Framework Directive (WFD) for the period 2012–2017 for 276 WFD assessment areas of varying size. Ecological status of an assessment area is assessed as high, good, moderate, poor, or bad using several indicators, one of which is the lower growth limit of continuous *Fucus* belt (Aroviita et al. 2019; ELY centres 2022).

### Effects of particulate accumulated matter on *Fucus*

We used species distribution modeling to assess whether the amount of PAM in the fluffy surface layer would affect the occurrence probability of *Fucus*. We fitted binomial generalized additive (GAM) models (Hastie and Tibshirani 1990) using R package mgcv (Wood 2011) with logit link using maximum likelihood estimation. Several environmental variables, previously used in similar modeling efforts, were used as predictors (Virtanen et al. 2018). The variable set was selected using background knowledge on factors potentially important in explaining *Fucus* distribution and removing strongly collinear variables (|r|> 0.7) (Dormann et al. 2013). The environmental variables used were observed abundance of PAM (factor variable with four levels, 0–3), measured depth, substrate class, mean seafloor fetch, seafloor slope, mean surface salinity, distance to sandy shore, total nitrogen, relative probability of reedbed (Koponen et al. 2022), share of sea, turbidity, and topographical shelter index (TSI). See previous section and Virtanen et al. (2024) for more detailed description of variables.

*Fucus* cover observations were converted to presence–absence by considering all occurrence observations as presences. We kept observations that were collected by diving, aquascope, or underwater video (see Forsblom et al., (2024) for a detailed description of the data). To ease the interpretation of the results, we restricted our occurrence analyses to depths between the surface and 30 m. This is well beyond the maximum growing depth of *Fucus*, which is 5–6 m in Gulf of Finland (Bäck and Ruuskanen 2000). The data consisted of 101 805 observations, of which 10 284 were presences. Prevalence of *Fucus* occurrence observations in the data was 0.101, 70% of the data was used to train the models, and 30% was retained for model validation. Similar prevalence of *Fucus* occurrence was maintained in both the training and validation sets.

Two candidate models were tested: a model with and without the PAM effect. Nested models were compared with Akaike Information Criteria (AIC). Model concurvity and convergence were checked with “gam.check” and “concurvity” functions from the mgcv package. Diagnostic plots were examined using Dunn–Smyth residuals with R package DHARMa (Hartig 2024). The simulated residuals were plotted against fitted values and all continuous and categorical explanatory variables. To quantify model predictive abilities, Tjur’s R^2^ and AUC values were calculated on the 30% data retained for model testing. Tjur’s R^2^ measures calibration of a binary classifier, i.e., how close the predicted probabilities aggregate around 0 and 1 on predicted absences and presences, respectively (Tjur 2009). AUC is a rank discrimination statistic, indicating how well the predicted probabilities discriminate between presences and absences (Lawson et al. 2014).

## Results

### Particulate accumulated matter model

**Fig. 2:**
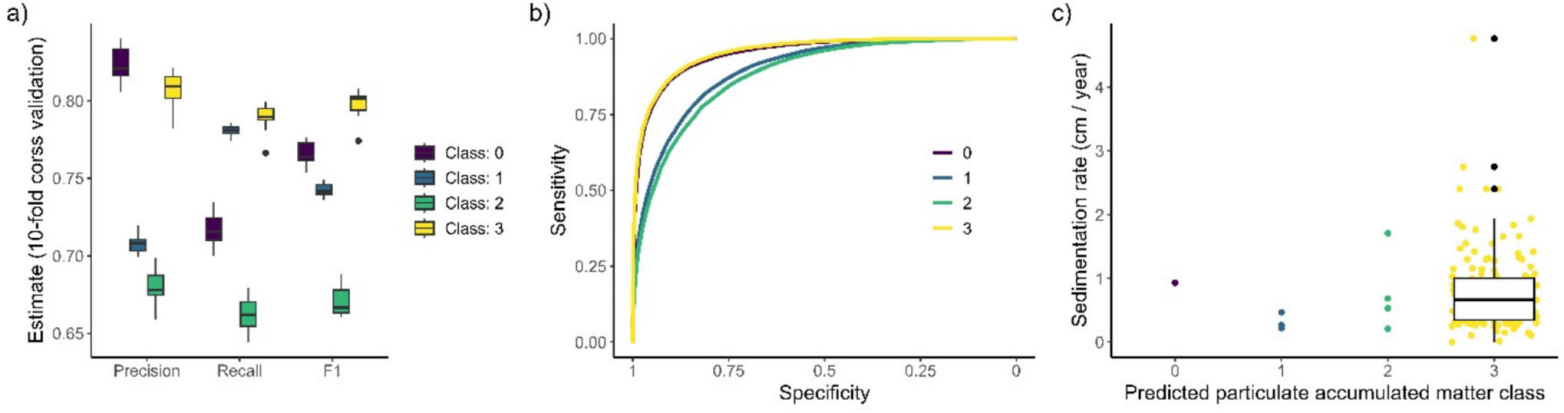
**a** Precision, recall, and F1 score for all PAM classes, from no accumulated particulate matter in the fluffy surface layer to very abundant accumulation (0–3), as well as **b** ROC curves for each class, all based on the tenfold cross-validation results. **c** Independent data on sedimentation rate depicting amount of settling sediment in the water column shown against the predicted particulate accumulated matter classes from the same location. Class 3 data are visualized with a jitter plot to support visualization

The mean overall model accuracy and kappa for the model were 0.74 and 0.64 based on the tenfold cross-validation. Looking at the class-specific score the model had good precision with over 0.8 for the lowest and highest PAM classes (Fig. 2a). The recall was best for the intermediate amount of PAM, class 2, and substantial amount of PAM, class 3. Looking at the F1 score, PAM class 2 was the hardest to classify correctly. The receiver operating characteristic curve also indicates that class 0 and 3, with no and substantial amounts of PAM, are easier to classify than the intermediate classes (Fig. 2b).

Comparison to data on sedimentation rates from sediment point data (Fig. 2c) was hampered by the low number of observations in areas predicted to have other than very abundant PAM (class 3). This is likely due to the fact that most data points on sedimentation rates were collected in deeper areas, and further offshore compared to areas identified as having no or little PAM. Also, the sedimentation rate dataset itself is biased to areas with high sedimentation rates. However, almost all observations where the sedimentation rates exceeded 1 were observed in areas where the predicted PAM was very high (class 3), with the exception of one observation, where the deposit was second highest.

**Fig. 3:**
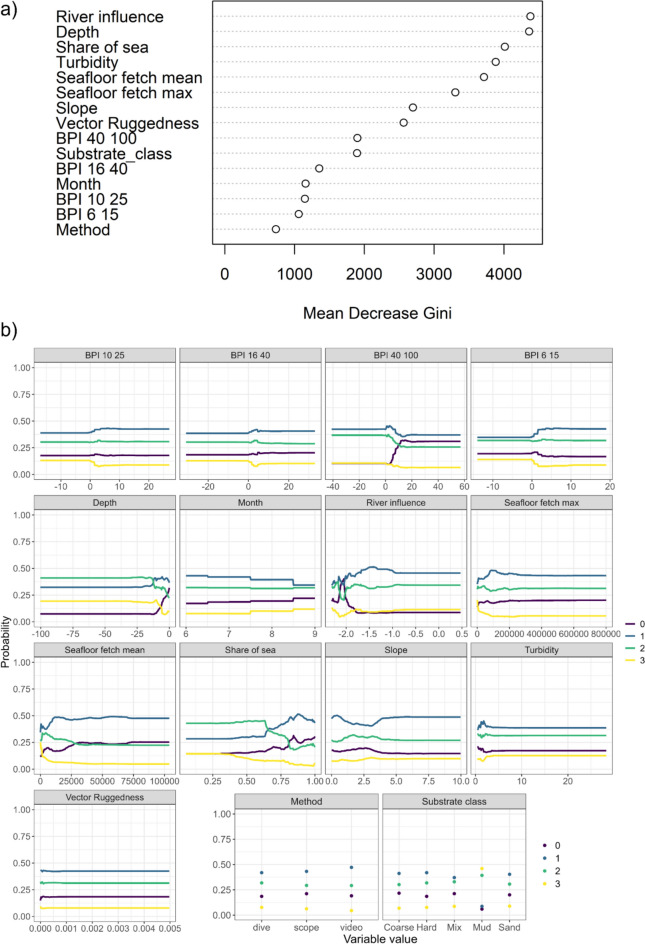
**a** Relative importance of environmental predictors measured as the mean decrease in Gini importance where large decrease indicates higher importance, **b** and partial predicted effects of each explanatory variable for all particulate accumulated matter classes with the other variables held at their mean. (BPI, Bathymetric Position Index, the first number describes the inner search radius and the second one outer)

The most influential variables explaining the distribution of PAM were river influence, depth, share of sea, and turbidity (Fig. 3). The partial probability for classes 1–3 increased with depth, whereas the no accumulation class increased in shallow areas. The partial effects of the share of sea indicated opposite patterns for the highest and the no accumulation classes, with higher probability for less deposition in exposed areas. For river influence all the classes with accumulation decrease as the distance to rivers increases. The class with no accumulation first increases with distance from the river but later increases again; this area corresponds to the majority of the observations for all classes (Fig. S1). There were very little differences in the levels of accumulation on different substrates and little differences between observation methods, indicating that PAM is a prevalent pressure in seafloor across habitat types (Fig. 3). We can also observe a slight increase of accumulation class 3 toward the late summer months, which could reflect increased accumulation after the late summer algal blooms. This is however hard to confirm, as there are no repeated visits to the sites.

### Projected amount and spatial distribution of PAM

**Fig. 4:**
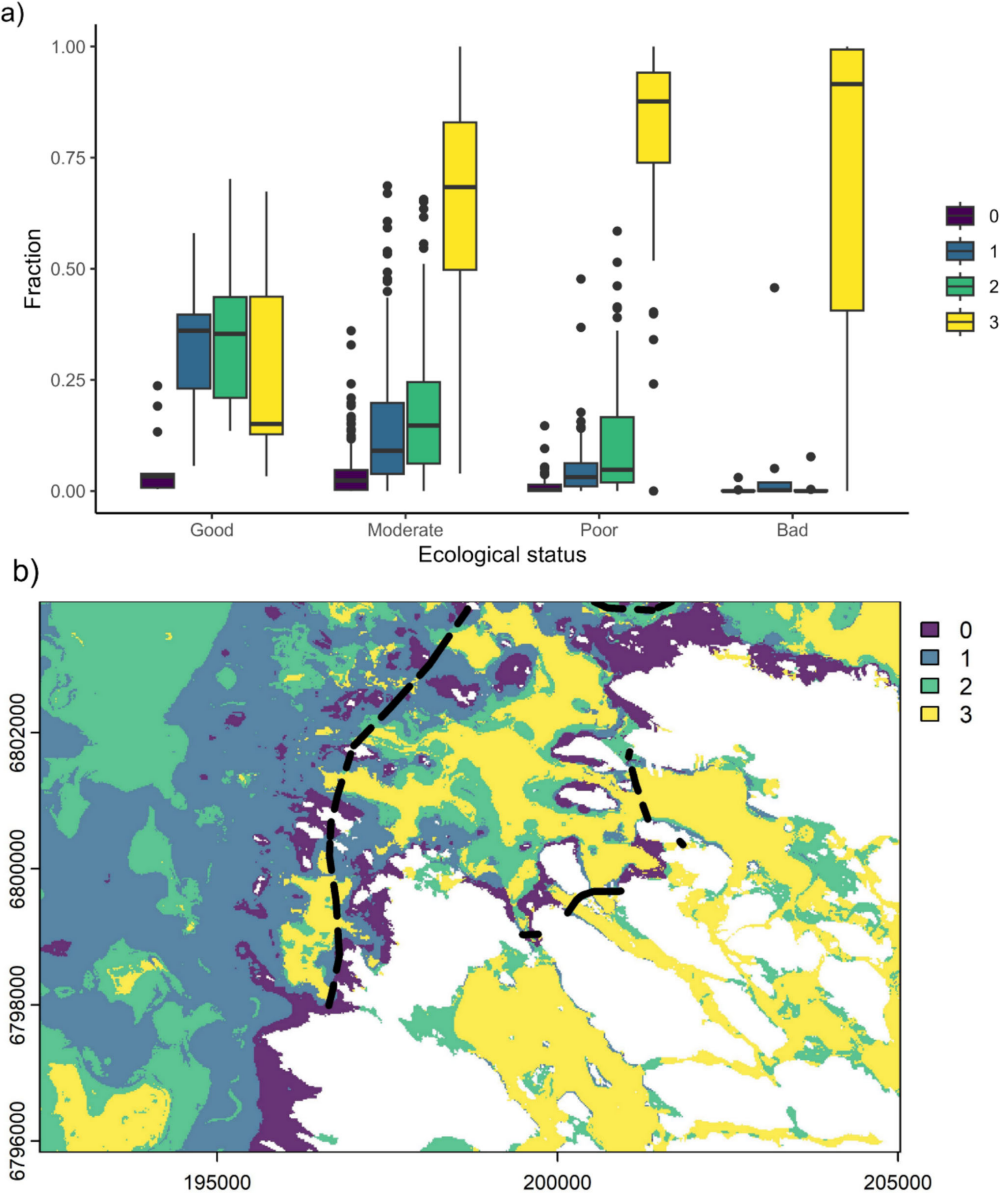
**a** Area percentage covered by the PAM classes in relation to the ecological status classification of 276 WFD areas. **b** Example of particulate accumulated matter prediction in a coastal area in the Bothnian Sea. Land shown in white and WFD borders shown in black

Based on predicted distribution of PAM classes on surfaces down to a depth of 25 m, a large fraction (36%) of the Finnish seafloor was covered by substantial amount (class 3) of surface accumulation. Only 4.8% remained without any kind of accumulation. Based on the predictions, many of the inner costal lagoons and more complex areas in the inner archipelago had substantial cover of PAM. Intermediate accumulation was prevalent in all areas, with classes 1 and 2 covering 28% and 31% of the seafloor, respectively (for full map see Fig. S2). We also compared the predicted PAM class with assessed ecological status defined by the WFD. The WFD status assessment areas classified as having poor, bad, and moderate status were mostly covered by substantial amount of PAM (level 3), whereas lower abundances were observed in areas classified as having good status (Fig. 4A). Areas with no accumulation (class 0) can mostly be found in more exposed areas along the open coast of islands (Fig. 4B and Fig. S2).

### Ecological relevance of particulate accumulated matter layer

PAM had a negative effect on *Fucus* occurrence. The GAM model with the PAM main effect had lower AIC than the model without PAM deposition (ΔAIC -343, Table 1). When evaluated with the 30% of the data retained for testing, inclusion of PAM improved model calibration (measured as Tjur’s R^2^), and discrimination ability (AUC). More details on model validation are given in Appendix S1.

**Table 1:** Tjur’s R^2^, AUC (on 30% test data), AIC values, and AIC difference of two fitted GAM models

Model	Tjur’s R^2^	AUC	AIC	ΔAIC
Null model	0.395	0.942	24 506	
PAM main effect	0.407	0.945	24 163	− 343.09

**Fig. 5:**
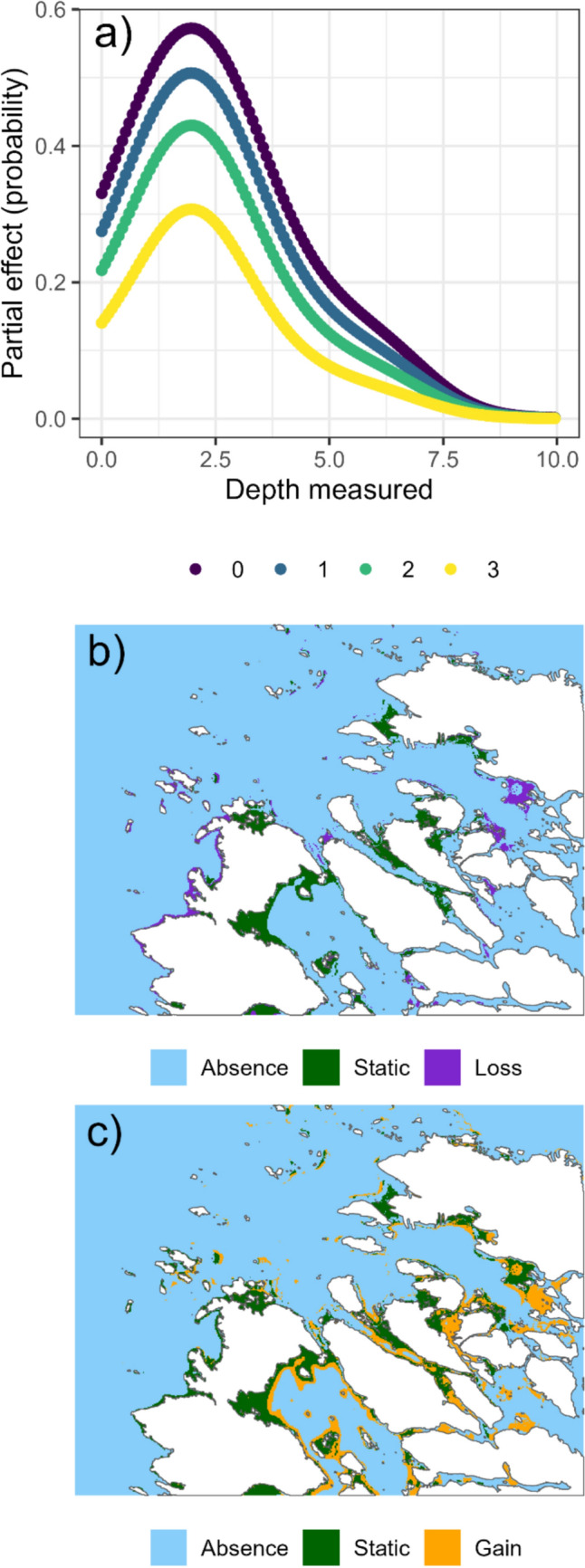
**a** The partial effect of depth on *Fucus* occurrence on hard substrates for each level of PAM, the current modeled *Fucus* habitat with **b** the expected loss in the scenario under high accumulation of particulate matter (level set to 3), and **c** the expected gain in the scenario with no accumulation (level set to 0). Habitat with no change shown as green. All other variables were kept at their mean values for the partial effect and habitat simulations

Increasing PAM lowered the occurrence probability of *Fucus* especially on hard substrates in the depth range 0–10 m (Fig. 5A), the occurrence decline being especially severe in the 2 to 3 m depth range, which is the known main occurrence depth range in Finnish coasts (Bäck and Ruuskanen 2000; Rinne and Salovius-Laurén 2020).

The effect of PAM on *Fucus* occurrence was mapped in a test area on the coast of the Bothnian Sea, northern Baltic Sea (for predicted PAM conditions, see Fig. 4). Under current conditions, the size of predicted *Fucus* habitat in the test area was 3.68 km^2^ (3.6% of total area, Fig. 5B). Extreme eutrophication, here considered as the substantial amount of PAM (class 3), would cause shrinkage of *Fucus* habitat to 2.41 km^2^ in the test area. This represents a loss of 34% compared to current situation, with majority of the loss of suitable habitat occurring in the more sheltered inner archipelago (Fig. 5C). In contrast, reducing eutrophication, here considered as no accumulation of particulate matter (setting class as 0), would result in almost doubling (7.16 km^2^, a 95% increase) the suitable *Fucus* habitat (Fig. 5D).

## Discussion

Here we demonstrate how to assess the ecological condition of benthic habitats using PAM accumulation as an indicator of localized eutrophication pressure. Only 4.8% of the shallow sea area was without PAM on the surfaces, which supports the assumption by Danielsson et al. 2007 that fluffy layer is assumed present all over the basin. We showed that using the fine scale benthic assessment approach applied here, more complex patterns emerge compared to the ecological status assessment of the WFD, particularly showing that areas assessed to be in moderate condition by the WFD have high fractions of PAM. This can be explained by the fact that the amount of PAM is likely governed by local conditions, for instance how exposed or sheltered an area is, and by the eutrophication gradient. This is supported by our results showing that areas with no PAM mainly occur in the most exposed shallow areas, whereas high PAM areas occur both in sheltered inner bays and in the open sea areas, where deeper accumulation bottoms occur. Thus, the PAM assessment increases local detail and could help designating locally meaningful mitigation and restoration measures. Using more local indicators of habitat condition, where PAM is jointly considered with the accumulation of epiphytic algae, has also been suggested to aid other local measures, such as biodiversity offsetting (Jalkanen 2025; Jalkanen et al. 2025). In ecological offsetting, harm caused to biodiversity in one area is compensated by enhancing nature somewhere else, and detailed knowledge of local variation in habitat condition is necessary. The indicators were developed together with national experts, and in that context, all PAM classes, except for the class with no accumulation, are considered harmful for benthic habitats (Jalkanen 2025). The present study enables spatially quantifying PAM and thus makes it possible to identify areas where habitat types could be at risk due to, e.g., burial, even if it is not possible to identify the exact source or composition of PAM based on our approach.

The areas predicted to have highest abundance of PAM were mostly the same as the areas that had the highest sedimentation rates when compared to independent data. The methods are not completely comparable, and the variability seen in the high PAM class might be due to wave action washing surfaces clean of PAM, even in areas where sedimentation rates might be high. Obviously, some areas are likely less sensitive to accumulation of particulate matter due to their wind and wave conditions. Danielsson et al., (2007) also found that the fluffy layers in most areas were resuspended at least once annually even if the underlying sediments are left unaffected. Especially enclosed areas, such as inner bays and lagoons, had high levels of PAM, highlighting their importance as accumulation areas and carbon sinks (Wikström et al. 2025).

The intermediate classes were much more unreliable and harder to model spatially, especially the intermediate PAM class 2. The accuracy of the PAM model might have been hampered by the scale of the environmental predictors, whereas depth and substrate were measured in situ together with PAM; the other environmental variables are based on modeled products at a resolution of 20 × 20 m grids. The challenge with distinguishing between the classes might also depend on the nature of the data, as visually identifying the absence of PAM and the most extreme case might be easier to standardize between observers, then the more nuanced differences between classes 1 and 2. This detectability influences our confidence in the spatial patterns of the intermediate classes. However, when considered at the observation level the response of *Fucus* was clear (Fig. 5A), with increasing PAM influencing the occurrence negatively, suggesting that for this purpose at in situ observation level the assessment of PAM classes worked.

Abundance of loose sediment has been suggested to be one driver for the disappearance of *Fucus* on deeper substrates, where wave action does not keep surfaces clean of sediment (Eriksson and Bergström 2005). Occurrence modeling results show that the amount of PAM affects the spatial distribution of *Fucus*. This interpretation is supported by previous experimental and field studies which provide mechanistic explanation for the observed pattern. The attachment of *Fucus* juveniles in size classes below 5 mm is substantially hindered if the hard settlement surfaces are covered with loose settled material (Eriksson and Johansson 2003, 2005). Even relatively low concentrations of surface sediment (0.1 g dm^−2^) cause very low survival rates of *Fucus* juveniles (Berger et al. 2004). This concurs with our results, which show that PAM affects the distribution of *Fucus* habitats and has merit as a potential indicator describing condition of benthic habitats. Increasing eutrophication pressure would most likely cause *Fucus* habitat to shift toward more exposed, shallow rocky shores, with sufficient wave action to keep the surfaces clear from PAM.

As the purpose of the PAM scenarios investigated (PAM classes 0 or 3) was to demonstrate the effect of PAM on *Fucus* distribution, we did not manipulate other model input variables, which would be sensitive to eutrophication, such as total nitrogen and turbidity. In a real setting, these would be affected by increasing eutrophication as well, potentially increasing the rate of habitat loss. It is also worthy to note that our test area was relatively shallow, having relatively large areas of hard substrates suitable for *Fucus*. Similar increases in suitable habitat by declining eutrophication pressure may not occur in all coastal areas due to lack of suitable substrate (Lappalainen et al. 2019).

## Conclusion

Here we have shown the potential of a strictly defined qualitative metric of PAM, coupled with spatial modeling, in estimating the fine-resolution direct impacts of eutrophication on seafloor habitats, which have gone mostly undetected by previous assessments less targeted at benthic habitats. We suggest PAM to be used as a tool for assessing local habitat condition of benthic habitats. The indicator could support Water Framework Directive assessments to better account for eutrophication-induced local effects on macrophyte-dominated habitats. This is especially timely as the European Commission’s Nature Restoration Law calls for assessing the condition of habitats, focusing restoration on areas deemed in poor condition. Our study shows that PAM has direct impact on *Fucus*, which is also most likely the case for other macroalgae and sessile invertebrate species, such as the blue mussel *Mytilus* spp., which also inhabit hard substrate bottoms. To develop the PAM indicator further, future studies should look more closely into the response of other species, including algae, submerged vegetation, and fauna, to PAM.

## Supplementary Information

Below is the link to the electronic supplementary material.

Supplementary file1 (PDF 586 kb)
