# Supplementary material for: Particulate accumulated matter as an indicator of coastal benthic habitat condition
Source: Ambio. 2025 Oct 11;55(4):891–902. doi: 10.1007/s13280-025-02249-y (PMC12960900; doi:10.1007/s13280-025-02249-y)
Supplement: Supplementary file 1 — Supplementary file1 (PDF 586 kb) [file 13280_2025_2249_MOESM1_ESM.pdf]

Supplementary Information:
Particulate accumulated matter as an indicator of coastal benthic habitat condition

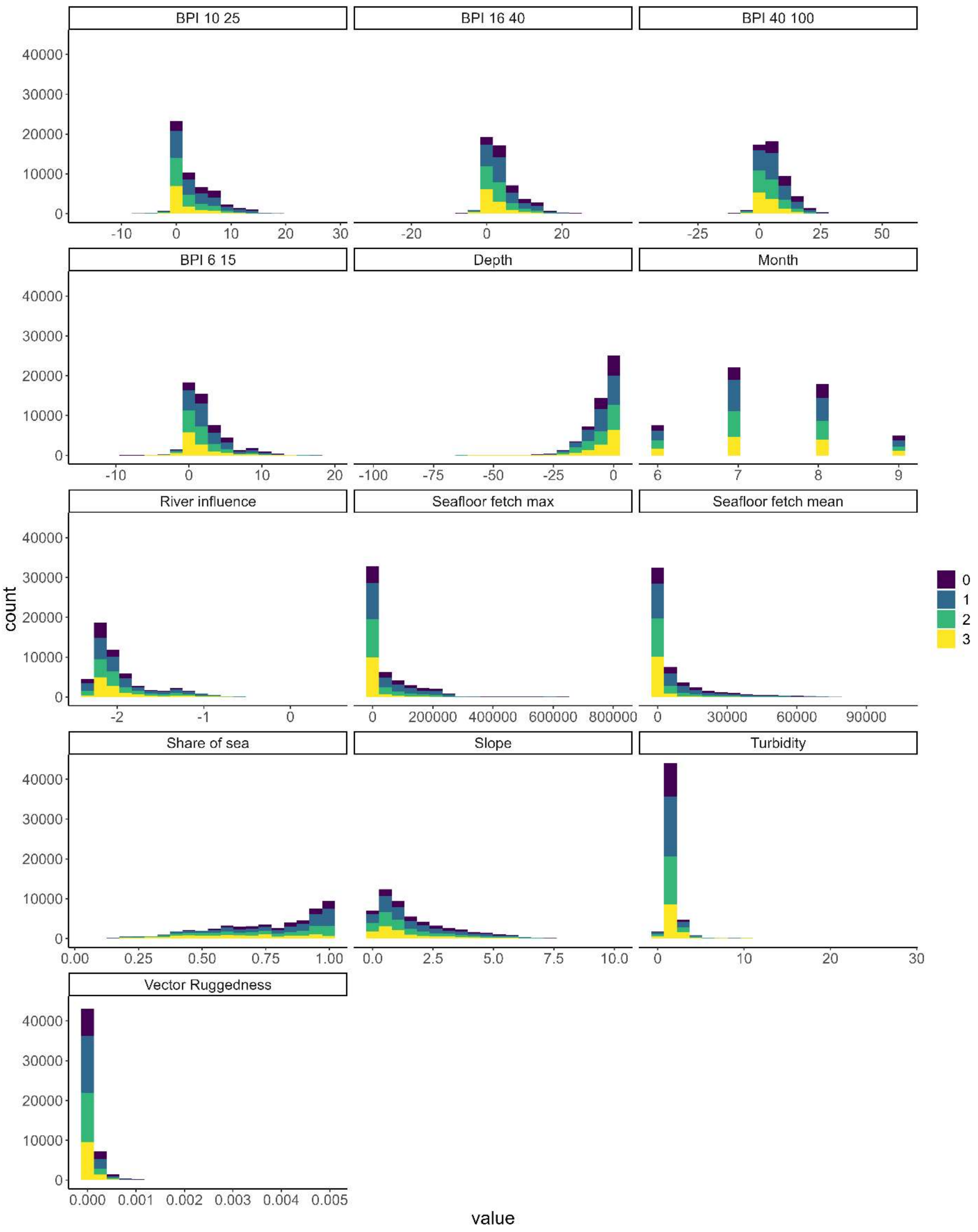

Fig S1. Histograms showing the distribution of the explanatory variables for each of the particulate accumulated matter classes (colors). (BPI = Bathymetric Position Index).

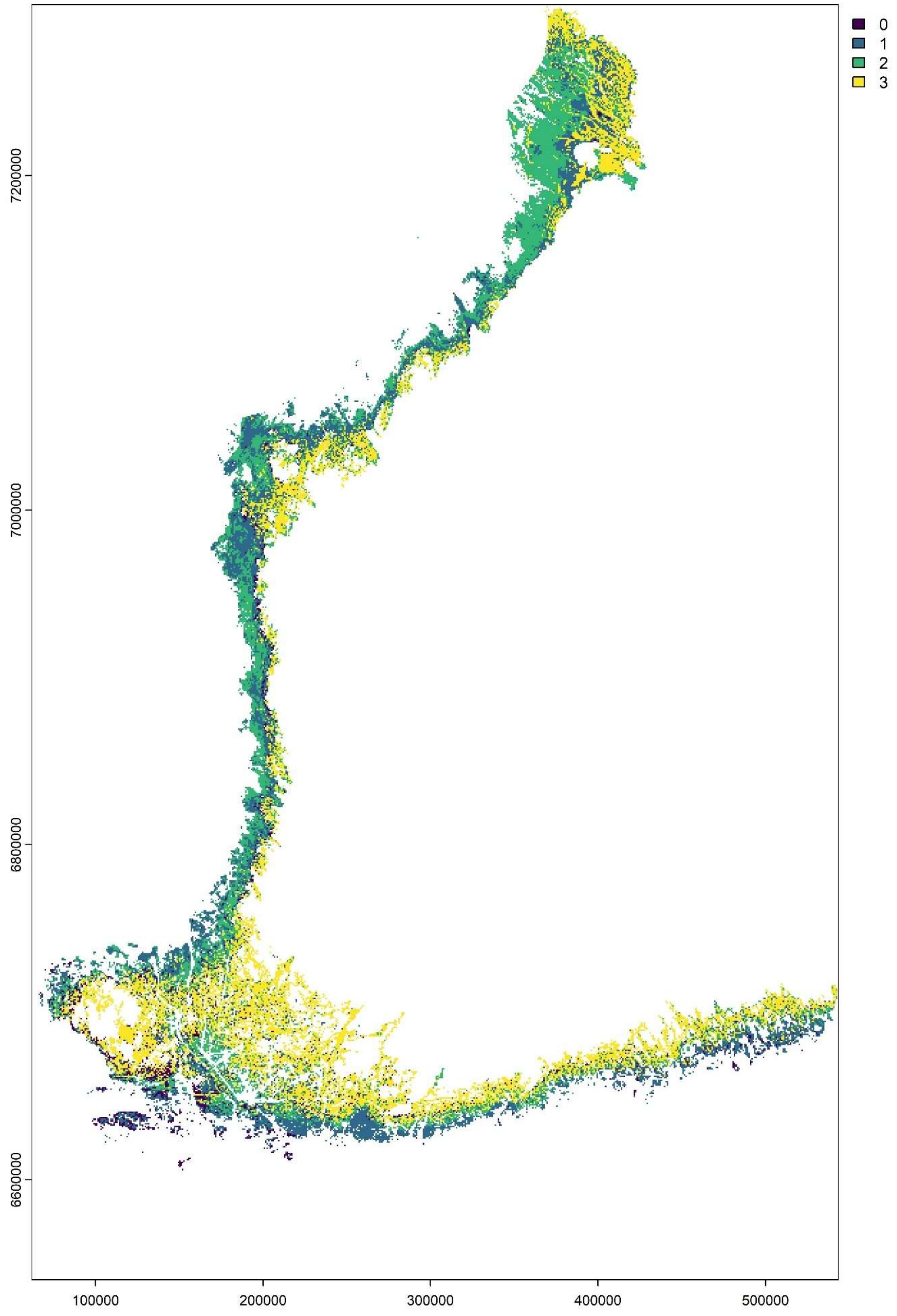

Fig. S2. Predicted class of particulate accumulated matter in the Finnish sea area down to a depth of 25 meters. Land and cropped deep areas shown in white.
